# Impact of different levels of zinc and nitrogen on growth, productivity, and quality of aromatic rice cultivated under various irrigation regimes in two districts of Pakistan

**DOI:** 10.3389/fpls.2022.951565

**Published:** 2022-07-26

**Authors:** Zuhair Hasnain, Shahbaz Khan, Fareeha Nasrullah, Kashf Mehmood, Danish Ibrar, Saqib Bashir, Ali Bakhsh, Irum Aziz, Afroz Rais, Naila Farooq, Sohail Irshad, Nabila Rashid, Jawaher Alkahtani, Mohamed S. Elshikh

**Affiliations:** ^1^Department of Agronomy, PMAS-Arid Agriculture University, Rawalpindi, Pakistan; ^2^National Agricultural Research Centre, Islamabad, Pakistan; ^3^Department of Botany, University of Agriculture, Faisalabad, Pakistan; ^4^Department of Biological Sciences, Superior University, Lahore, Pakistan; ^5^Department of Soil & Environmental Science, Ghazi University, Dera Ghazi Khan, Pakistan; ^6^Department of Plant Breeding and Genetics, Ghazi University of Agriculture, Dera Ghazi Khan, Pakistan; ^7^Department of Botany, Sardar Bahadur Khan Women’s University, Quetta, Pakistan; ^8^Department of Soil & Environmental Sciences, College of Agriculture, University of Sargodha, Sargodha, Pakistan; ^9^Department of Agronomy, MNS-University of Agriculture, Multan, Pakistan; ^10^Department of Botany and Microbiology, College of Science, King Saud University, Riyadh, Saudi Arabia

**Keywords:** irrigation, kernel, nutrient, net assimilation, paddy, radiation, resources

## Abstract

Rice is a staple food for more than 50% of the global population and it is one of the most valuable cereal crops. To fulfill the dietary requirement of the ever-growing world population, an increase in per-unit production of rice is direly required. In Pakistan, it stands as the 2nd in consumption after wheat, which is a staple food. A huge gap is observed between yield potential and actual yield of the aromatic rice cultivars at a farmer-field level. The significant limitations responsible for this gap are shortage of irrigation water, inappropriate application of fertilizers, less plant population, deficiency of micronutrients, and improper and poor plant protection measures. A field study was planned to assess the yield response and quality attributes of aromatic rice to three levels of zinc (Zn) and nitrogen (N) under three irrigation regimes (8-, 12-, and 16-acre inches) in the Sheikhupura and Sargodha districts of Pakistan. Irrigation treatments significantly influenced the growth, yield, and quality attributes; however, maximum improvement was observed by the application of irrigation at 12-acre inches. Among the Zn treatments, application of Zn at 10 kg ha^–1^ was observed to be more responsive to improving the growth and quality parameters of aromatic rice crops. In the case of N treatments, application of N at 140 kg ha^–1^ produced the maximum total tillers, as well as productive tillers per hill, spikelets per panicle, leaf area index, leaf area duration, crop growth rate, total dry matter, harvest index, kernel length, kernel width, and 1,000-kernel weight. Application of N at 140 kg ha^–1^ not only improved the growth attributes but also increased the net assimilation rate, photosynthetically active radiation, and radiation use efficiency, with respect to total dry matter and kernel yield. The maximum percentage of normal kernels and minimum percentage of opaque, abortive, and chalky kernels were also recorded by application of N at 140 kg ha^–1^. The outcomes of current experiments depicted that application of irrigational water, zinc, and nitrogen at 12-acre inches, 10, and 140 kg ha^–1^, respectively, are responsible to achieve maximum resource utilization efficiency, along with increased yield and quality of rice.

## Introduction

Rice is a prominent cereal crop and staple food of masses around the globe. Among agricultural commodities, its cultivation requires an ample quantity of water. In Pakistan, it is the second most-consumed food after wheat. In 2021, it was cultivated on an area of 3,335 thousand hectares with a production of 8.419 million tonnes (Government of Pakistan, 2021). For the cooking purposes, aromatic rice (basmati) is mainly preferred because of its long fluffy grains and unique taste. Least kernel dimension, aroma, fine texture, maximum elongation the of kernel during cooking, and high palatability with longer shelf life are some of the dynamic features of basmati rice (Ahmad et al., 2005). New basmati varieties, such as basmati super, basmati-2000, and shaheen basmati developed through different breeding approaches, have higher yield potential and improved quality characteristics. There is a huge yield gap between the potential yield and the actual yield of rice cultivars attained by the farmers. The potential yield of various basmati cultivars ranges from 4.5 to 6-ton ha^–1^ while farmers are getting only 2–2.8-ton ha^–1^, which is quite low (Ahmad et al., 2005; Shivay et al., 2010). Rice straw is commonly considered an agricultural waste that is a good source of sugar (Singhajutha et al., 2020). Pakistan is earning foreign exchange by exporting aromatic rice despite its actual yield being quite low than its genetic potential. Therefore, it is a timely need to strengthen research and planning to improve food production and quality to meet the standards of the international market.

The yield of basmati rice is low because of many factors, including water shortage, less plant population, non-judicious use of chemical fertilizers, zinc (Zn) deficiency, and poor plant protection measures. Among these factors, deficiency of water irrigation (Xie et al., 2008), nitrogen (N) fertilizer management (Wang et al., 2008), deficiency of Zn (Shivay et al., 2010), and weed infestation (Hossain et al., 2021a) have crucial effects to the growth, yield, and quality attributes of aromatic rice. Excess or deficiency of any input limits the growth and yield of field crops. Worldwide, water scarcity is a burning issue these days and sufficient irrigation of water supply is among the important factors for enhancing the yield of field crops (Xie et al., 2008). Prasad et al. (2008) reported that irrigation management had a significant impact on the growth, yield, and quality of rice. Sharma et al. (2008) reported the significant effects of irrigation regimes on grain, straw yield, and nutrient concentration of rice. There was a synergistic effect between irrigation levels and growth, quality, and nutrient concentration of upland rice (Crusciol et al., 2003).

Zinc (Zn) is an important micronutrient with vital roles in rice growth and metabolism. Its deficiency is a major concern in achieving target yield of field crops. Sarwar et al. (2020) and Anwar et al. (2022) reported that mineral fertilization, particularly micronutrients (Zn and Fe), is responsible for improved crop productivity and enhanced quality (Hussan et al., 2022). Its deficiency is mainly reported in rice-growing areas worldwide, including Pakistan. Paddy yield is reduced (25–50%) because of its deficiency, therefore, it is imperative to apply Zn in adequate quantity and at the proper time to attain maximum yield of rice and vegetable crops as well (Zafar et al., 2022). Mirzavand (2007) reported that an adequate supply of Zn to rice crops resulted in higher protein contents in grain. In Asian countries, it is a major health risk factor where rice is a staple food, and Zn nutrition for human and animal health has recently attained significant attention (WHO, 2002). Little work has been carried out and less information is available on the impact of Zn fertilization on kernel quality of basmati rice in Pakistan (Khan et al., 2009).

In modern agriculture, intensive cultivation is mandatory to meet the demands of ever-increasing population. In this scenario, N is dynamic macronutrient that has major role in crop production and improves the defensive system under competitive environment (Imran et al., 2021; Huang et al., 2022). Wang et al. (2008) reported a significant increase in the number of tillers and panicles per plant, photosynthetic rate, chlorophyll contents, and grain yield with an increased supply of N to rice and wheat crops (Wang et al., 2022). An adequate supply of N to rice crops resulted in an increased concentration of protein (Singh and Tripathi, 2007). A wealth of literature is available, depicting an increase in rice yield with increased N concentration to a certain level, while an N with very high rate resulted in low yield and N utilization by the rice. According to Ren et al. (2020), the difference in rice yield is mainly because of the difference between seed setting and several effective panicles. Meng and Du (2013) showed that an appropriate N application rate could ensure that the rice canopy population reached a higher leaf area index. It was investigated in a study of rice that nitrogenous fertilizer, amended with bacteria, was responsible for the variation in the various biochemical activities (Fatma et al., 2021). Breeding techniques are also under consideration in developing new varieties of field crops, particularly rice (Darwish et al., 2021; Herawati et al., 2021; Rahman et al., 2021), with respect to enhanced nitrogen use efficiency (Javed et al., 2022a,b). Various management approaches are being adopted by the farmers’ community to enhance the productivity of agronomic and horticultural crops (Khasanah and Rachmawati, 2020; Hossain M. A. et al., 2021b; Rehman et al., 2021), including the intercropping, application mineral elements, synthetic compounds (Nurhidayati et al., 2020; Abello et al., 2021), organic amendments (Iqbal et al., 2020; Tabaxi et al., 2021), plants extracts, and biostimulants (Makawita et al., 2021; Khan et al., 2022) *via* soil (Rahim et al., 2020), seed coating (Javed et al., 2021), seed priming agents, and foliar spray (Khan et al., 2017; Batool et al., 2019; Farooq et al., 2021). These practices are not only responsible for productivity enhancement, but they significantly mitigate the adverse impacts of abiotic and biotic stresses (Shareef, 2020). It was hypothesized that the integrated application of N and Zn may enhance the productivity and quality of rice crops. In the light of rationale, the current experiment was designed to explore the impact of various levels of N along with Zn application on the growth, production, and quality of aromatic rice crop cultivated under various irrigation regimes at diverse agro-environmental conditions in Pakistan.

## Materials and methods

### Experimental particulars and crop husbandry

**FIGURE 1:**
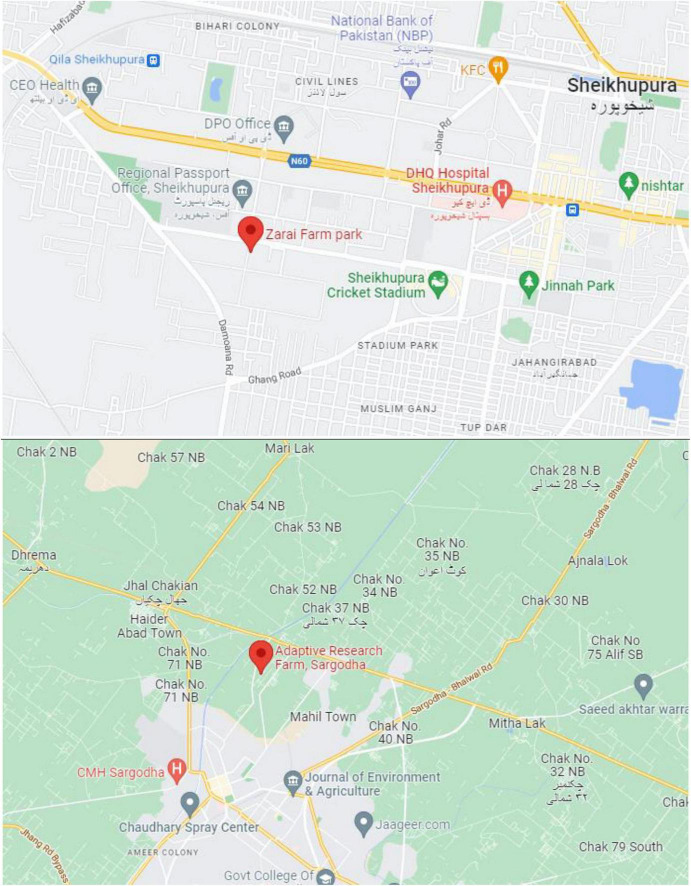
Geographical map of the study area: Adaptive research farm, Sheikhupura (Zarai Farm Park) and adaptive research arm, Sargodha.

The Adaptive Research Farm, Sheikhupura (31.6°N, 74.6°E, and 217 m asl) and Adaptive Research Farm, Sargodha (32.04°N, 72.67°E and 188 m asl) were selected to conduct the designed experiments during the rice cultivation season. Geographical maps of the study areas are given in Figure 1. Aromatic rice nursery was sown after 20th May and nursery was transplanted after 40 days of nursery sowing. A recommended seed rate of 10 kg per ha was used to raise the nursery. For raising the nursery, the wet bed method was used. For this purpose, on the pulverized soil, rice seeds were spread manually. Regarding fertilization of rice nursery, 1.5 kg/6 marlas of N was applied. Super Basmati cultivar of aromatic rice was selected to observe the impact of treatments regarding growth, yield, and quality traits. Before, conducting the experiment, composite soil samples were collected from the study area with the help of an auger up to the depth of 30 cm. Physical and chemical properties of the experimental soils are presented in Table 1. Before transplanting, at the time of puddling, required doses of fertilizers applied to fulfill the nutrient required of rice crop. Urea, single superphosphate, potassium sulfate, and zinc sulfate were used as the sources of nutrients. Half dose of nitrogen was applied at the puddling, remaining half was applied at the tillering stage of the rice crop for maximum utilization of applied fertilizer. Depths of irrigation water, i.e., 8-, 12-, and 16-acre inches, were measured by installing the cut-throat flume at the field channel and calculations were made as described by Boman and Shukla (2006). To avoid the seepage effect or boarder impact, buffers plants were designed and maintained throughout the course of experimentation. Agronomic practices, including weeding and plant protection measures, etc., were uniformly performed during the growth period of rice crop. Flooded conditions were also observed regularly and maintained during the study. In the current experimentation, three factors, i.e., irrigation, zinc, and nitrogen, were under study with three levels of each factor (Irrigation; 8, 12, and 16-acre inches, Zn; 5, 10, and 15 kg ha^–1^, and N; 70, 140, and 210 kg ha^–1^).

**TABLE 1:** Physical and chemical characteristics of experimental soil.

Determination	Unites	Sheikhupura	Sargodha
**Physical characteristics**			
Sand	%	14	23
Silt	%	70	60
Clay	%	16	17
Texture class		Loam	Silty loam
**Chemical characteristics**			
pH		8.4	7.6
Total soluble salts	%	10.1	15.02
Organic matter	%	0.80	0.96
Total nitrogen	%	0.07	0.06
Available phosphorus	ppm	10.4	16.80
Available potassium	ppm	204	235

### Measurement of agronomic yield and quality attributes

Before the harvesting of crop, five hills were tagged randomly in each experimental unit to collect the data regarding total number of tillers per hill, number of panicle-bearing tillers, spikelet per panicle, and percentage of spikelet sterility. Total number of tillers per hill was counted manually from five tagged hills and the average was calculated. Tillers having panicles were separated and counted to record the number of panicle-bearing tillers (productive tillers). Number of spikelets per panicle was also counted manually from the productive tillers and the average was calculated. Spikelet sterility was recorded by observing the spikelet without grain and percentage was calculated. To record the data of yield and its components, an area of 7.5 m^2^ was selected and harvested manually. Before threshing, all the harvested stuff was sun-dried for a week. Harvest index was expressed in percentage after calculating the ratio of grain yield to total dry matter. Thousand (1,000) kernels were counted manually from each experimental unit and weighed by electric balance to record the 1,000-kernel weight. A digital caliper was used to record the dimensions (length and width) of kernels. Tillers were randomly selected from each experimental unit to observe normal, opaque, abortive, and chalky kernels according to the procedure described by Nagato and Chaudhry (1969).

### Measurement of growth parameters

Destructive sampling was done to record the data related to leaf area index, leaf area duration, crop growth rate, net assimilation rate, and photosynthetically active radiation. With an interval of fortnightly duration, four plants were harvested at ground level from each experimental unit to record the data regarding growth attributes. Fresh and dry weights of all parts of plant (stem, leaves, and panicle) were recorded. To record the dry weight, samples were dried in oven at 70°C up to a constant weight. Leaf area was recorded according to the protocols of Watson (1952). Leaf area duration, crop growth rate, and net assimilation rate were measured according to the formulas described by Hunt (1978). Photosynthetically active radiations were measured by following the method of Szeicz (1974). Radiation use efficiency, with respect to total dry matter (RUE_TDM_) and grain yield (RUE_KY_), was calculated according to following equations:

$${\mathrm{RUE}}_{\mathrm{TDM}}=\mathrm{TDM}/\Sigma \,\mathrm{Sa}$$

and

$$R\,U\,E_{K\,Y}=K\,e\,r\,n\,e\,l\,y\,i\,e\,l\,d/\Sigma \,S\,a$$

Where Sa is amount of intercepted light.

### Statistical analysis

Normal data distribution assumption was tested using SPSS v. 17.0 software 195 packages according to the Shapiro and Wilk (1965) method The combined ANOVA was carried out according to Snedecor and Cochran (1994), to estimate the main effects of the different sources of variation and their interactions. *F*-test was used to test treatment significance at 5% probability level using “MSTAT-C” software package. Mean separation was done using least significant difference (LSD) test when significant differences were found (Steel et al., 1997).

## Results

Irrigation treatments significantly influenced the growth attributes, i.e., total number of tillers per hill, panicle bearing tillers per hill (productive tillers), spikelets per panicle, and spikelet sterility percentage (Table 2). Among all the irrigation treatments, application of irrigation at 12-acre inches produced maximum total tillers per hill, productive tillers per hill, and spikelets per panicle at both sowing places. Same treatment also reduced the spikelet sterility percentage as well (Table 2). Zn treatments significantly affected the total tillers per hill, productive tillers per hill, and spikelet sterility percentage except for spikelets per panicle. Application of Zn at 15 kg ha^–1^ was observed to be more responsive as compared to other treatments regarding growth attributes. N levels significantly influenced the growth parameters except for spikelet sterility percentage, and application of nitrogen at 140 kg ha^–1^ produced the maximum number of total tillers per hill, productive tillers per hill, and spikelets per panicle (Table 2). Interactive impact of irrigations, such as Zn and N applications, is also presented in the Table 2.

**TABLE 2:** Impact of zinc and nitrogen rates on number of tillers per hill, panicle bearing tillers, spikelets per panicle, and percentage of spikelet sterility of rice cultivated under three irrigation levels at Sheikhupura (D1) and Sargodha (D2) districts of Pakistan.

Parameters	Tillers per hill		Panicle bearing tillers		Spikelets per panicle		Percentage of spikelet sterility	
				
Treatments	D1	D2	D1	D2	D1	D2	D1	D2
**A = Irrigation levels**								
I_1_ = 8	14.73c	14.52b	13.80b	12.61b	163.28b	166.7b	6.93a	7.00a
I_1_ = 12	15.92a	15.98a	14.93a	13.94a	172.93a	173.8a	6.47b	6.34c
I_1_ = 16	15.28b	15.03ab	14.12b	13.38ab	170.41a	168.7b	6.60b	6.64b
SE	0.18	0.39	0.27	0.34	2.33	1.75	0.11	0.10
LSD	0.50*	1.08*	0.74*	0.93*	6.48*	4.86*	0.31*	0.29*
**B = Zinc rates**								
Zn_1_ = 5 kg ha^–1^	13.79b	13.46b	12.80b	12.22b	165.13	166.97	6.79a	6.76a
Zn_2_ = 10 kg ha^–1^	16.09a	16.11a	15.07a	13.97a	171.51	171.64	6.50b	6.46b
Zn_3_ = 15 kg ha^–1^	16.04a	15.97a	14.98a	13.73a	169.96	170.60	6.71a	6.74a
SE	0.15	0.17	0.16	0.20	2.54	1.94	0.06	0.04
LSD	0.34**	0.37**	0.36**	0.43**	NS	NS	0.14**	0.10**
**C = Nitrogen rates**								
N_1_ = 70 kg ha^–1^	15.13b	14.96b	14.12b	12.95c	164.72b	167.3b	6.78	6.72
N_2_ = 140 kg ha^–1^	15.49a	15.47a	14.49a	13.56a	172.85a	171.28a	6.55	6.58
N_3_ = 210 kg ha^–1^	15.3ab	15.11ab	14.25b	13.41b	169.04ab	170.66a	6.68	6.66
SE	0.13	0.20	0.12	0.06	2.35	1.23	0.07	0.06
LSD	0.27*	0.41*	0.24**	0.12**	4.76*	2.49*	NS	NS
**Interactive effect of irrigation (A), zinc (B), and nitrogen (C) rates**								
A × B	*	*	**	NS	NS	NS	NS	**
A × C	NS	NS	**	**	NS	NS	NS	NS
B × C	NS	NS	NS	**	NS	NS	NS	NS
A × B × C	NS	NS	**	*	NS	NS	NS	*

Means sharing the same alphabet within the column are not statistically significant.

SE, standard error; LSD, least significant difference value for comparison; NS, statistically non-significant.

* and ** Significant at P < 0.05 and 0.01, respectively.

Irrigation treatments significantly affected the harvest index, kernel length and width, and 1,000- kernel weight of rice cultivated under three irrigation levels (Table 3). Among all irrigation treatments, application of irrigation at 12-acre inches produced maximum harvest index, kernel length and width, and 1,000-kernel weight at both sowing regions. Zn treatments significantly affected the harvest index, kernel length and width, and 1000-kernel weight. Application of Zn at 10 kg ha^–1^ was observed more responsive as compared to other treatments. N levels also significantly improved these attributes and application of nitrogen at 140 kg ha^–1^ produced maximum harvest index, kernel length and width, and 1,000-kernel weight. Interactive impact of irrigations, such as Zn and N applications, is also presented in the Table 3.

**TABLE 3:** Impact of zinc and nitrogen rates on harvest index, kernel length, and width, and 1,000-kernel weight of rice cultivated under three irrigation levels at Sheikhupura (D1) and Sargodha (D2) districts of Pakistan.

Parameters	Harvest index		Kernel length		Kernel width		1000-kernel weight	
				
Treatments	D1	D2	D1	D2	D1	D2	D1	D2
**A = Irrigation levels**								
I_1_ = 8	25.79c	24.53b	6.43b	6.52b	1.65b	1.71b	16.63b	19.01b
I_1_ = 12	33.95a	32.18a	6.73a	6.88a	1.84a	1.96a	18.50a	20.37a
I_1_ = 16	30.25b	27.36b	6.54ab	6.76a	1.78a	1.80ab	17.64ab	19.71ab
SE	0.01	0.01	0.08	0.09	0.04	0.06	0.46	0.36
LSD	0.03**	0.04**	0.21*	0.24*	0.12*	0.17*	1.27*	1.00*
**B = Zinc rates**								
Zn_1_ = 5 kg ha^–1^	26.61b	25.16c	6.48b	6.52b	1.72b	1.75b	17.03b	19.43b
Zn_2_ = 10 kg ha^–1^	32.54a	30.93a	6.68a	6.89a	1.82a	1.90a	18.00a	19.83a
Zn_3_ = 15 kg ha^–1^	30.84a	27.97b	6.54ab	6.74a	1.73b	1.82ab	17.74a	19.82a
SE	0.006	0.005	0.07	0.09	0.03	0.06	0.32	0.24
LSD	1.85**	1.24**	0.14*	0.19**	NS	NS	0.70*	0.35*
**C = Nitrogen rates**								
N_1_ = 70 kg ha^–1^	28.67b	26.84b	6.47b	6.57b	1.71b	1.72c	17.15b	19.06c
N_2_ = 140 kg ha^–1^	31.13a	29.75a	6.67a	6.86a	1.79a	1.92a	17.99a	20.18a
N_3_ = 210 kg ha^–1^	30.19a	27.48b	6.57ab	6.72ab	1.76ab	1.84b	17.63ab	19.84b
SE	0.003	0.004	0.07	0.11	0.03	0.04	0.33	0.08
LSD	1.56**	1.93**	0.13*	0.23*	0.06*	0.08**	0.66*	0.16*
**Interactive effect of irrigation (A), zinc (B), and nitrogen (C) rates**								
A × B	NS	NS	**	NS	**	NS	NS	NS
A × C	NS	NS	NS	NS	NS	NS	NS	NS
B × C	NS	NS	NS	NS	NS	NS	NS	NS
A × B × C	NS	NS	NS	NS	NS	NS	NS	NS

Means sharing the same alphabet within the column are not statistically significant.

SE, standard error; LSD, least significant difference value for comparison; NS, statistically non-significant.

* and ** Significant at P < 0.05 and 0.01, respectively.

Irrigation treatments significantly influenced the percentage of normal, opaque, and abortive kernel of rice cultivated under three irrigation levels at Sheikhupura and Sargodha districts of Pakistan (Table 4). Among all the irrigation treatments, application of irrigation at 12-acre inches produced maximum percentage of normal kernel. Same treatment also reduced the opaque and abortive kernel, while maximum rate of opaque and abortive kernel was observed at irrigation level of 8-acre inches. Zn treatments also significantly affected these attributes. Application of Zn at 10 kg ha^–1^ improved the maximum percentage of normal kernel as compared to other treatments. Similarly, highest reduction in opaque and abortive kernel was also noted at 10 kg ha^–1^ Zn application level, while maximum rate of these parameters was observed at 5 kg ha^–1^ Zn application level. N levels significantly influenced the percentage of normal, opaque, and abortive kernel of rice, and application of nitrogen at 140 kg ha^–1^ produced maximum percentage of normal kernel. However, maximum reduction in rate of opaque and abortive kernel was also observed at 140 kg ha^–1^ nitrogen level, while highest opaque and abortive kernel rate was noted at 70 kg ha^–1^ nitrogen level. Chalkiness was not significantly influenced by the treatments. Interactive impact of irrigations, such as Zn and N applications, is also presented in the Table 4.

**TABLE 4:** Impact of zinc and nitrogen rates on percentage of normal, opaque, and abortive kernel of rice cultivated under three irrigation levels at Sheikhupura (D1) and Sargodha (D2) districts of Pakistan.

Parameters	Percentage of normal kernel		Percentage of opaque kernel		Percentage of abortive kernel		Percentage of chalky kernel	
				
Treatments	D1	D2	D1	D2	D1	D2	D1	D2
**A = Irrigation levels**								
I_1_ = 8	65.02b	71.82b	10.99a	12.35a	9.34a	10.98a	14.65	4.85
I_1_ = 12	69.02a	74.28a	10.03b	10.67c	8.65b	8.83b	12.3	6.22
I_1_ = 16	66.44b	73.66a	10.44b	11.48b	8.91ab	10.00ab	14.21	4.86
SE	0.82	0.65	0.19	0.23	0.18	0.57	0.23	0.14
LSD	2.29*	1.81*	0.52*	0.64*	0.50*	1.58*	NS	NS
**B = Zinc rates**								
Zn_1_ = 5 kg ha^–1^	66.04	72.11	11.27a	11.97a	9.34a	10.56a	13.35	5.36
Zn_2_ = 10 kg ha^–1^	67.87	73.97	9.49c	11.17b	8.47c	9.12c	14.17	5.74
Zn_3_ = 15 kg ha^–1^	66.58	73.68	10.70b	11.37b	9.09b	10.14b	13.63	4.81
SE	0.63	0.86	0.20	0.24	0.11	0.18	0.19	0.32
LSD	NS	NS	0.43**	0.52**	0.24**	0.38**	NS	NS
**C = Nitrogen rates**								
N_1_ = 70 kg ha^–1^	65.65b	72.15b	10.68a	11.79a	9.09a	10.20a	14.58	5.86
N_2_ = 140 kg ha^–1^	67.98a	74.37a	10.28c	11.26b	8.81b	9.75b	12.93	4.62
N_3_ = 210 kg ha^–1^	66.89ab	73.23b	10.49b	11.46b	9.00a	9.87b	13.62	5.44
SE	0.76	0.54	0.09	0.12	0.09	0.14	0.21	0.15
LSD	1.54*	1.09**	0.18**	0.25**	0.18*	0.29**	NS	NS
**Interactive effect of irrigation (A), zinc (B), and nitrogen (C) rates**								
A × B	**	NS	*	NS	*	NS	NS	NS
A × C	NS	NS	NS	NS	NS	NS	NS	NS
B × C	NS	NS	*	NS	NS	NS	NS	NS
A × B × C	NS	NS	*	NS	NS	NS	NS	NS

Means sharing the same alphabet within the column are not statistically significant.

SE, standard error; LSD, least significant difference value for comparison; NS, statistically non-significant.

* and ** Significant at P < 0.05 and 0.01, respectively.

Data related to leaf area index, leaf area duration, total dry matter, and crop growth rate are presented in Table 5. Among all irrigation levels, 12-acre inches irrigation attained highest values of leaf area index, leaf area duration, and total dry matter, while crop growth rate and minimum values of said attributes were observed in 8-acre inch irrigation. Zn application also significantly affected the aforementioned parameters. Zn application at 10 kg ha^–1^ was found more effective as compared to other Zn levels in relation to growth parameters (Table 5). N levels significantly affected growth attributes. The highest increment in all growth parameters presented in Table 5 was observed in the case of nitrogen at 140 kg ha^–1^ and the minimum values were observed at 70 kg ha^–1^ N that was supplemented at both sowing places. The interactive effect between irrigation levels and Zn and N rates was non-significant for leaf area duration and crop growth rate, while interaction between Zn and N levels in the case of leaf area index was significant at first sowing place, and interaction between irrigation levels and Zn application, while Zn and N application was highly significant at second sowing place. Interaction between irrigation levels and Zn application was highly significant at first sowing place in case of total dry matter (Table 5).

**TABLE 5:** Impact of zinc and nitrogen rates on leaf area index, leaf area duration, total dry matter and crop growth rate of rice cultivated under three irrigation levels at Sheikhupura (D1) and Sargodha (D2) districts of Pakistan.

Parameters	Leaf area index		Leaf area duration		Crop growth rate		Total dry matter	
				
Treatments	D1	D2	D1	D2	D1	D2	D1	D2
**A = Irrigation levels**								
I_1_ = 8	4.77b	5.21b	257.1b	282.9b	10.35b	10.60b	13.87c	14.12b
I_1_ = 12	5.34a	5.82a	285.0a	321.2a	11.29a	11.89a	15.35a	15.76a
I_1_ = 16	5.05ab	5.48ab	271.5ab	299.5ab	10.63b	11.45ab	14.98b	15.46a
SE	0.14	0.16	5.75	9.68	0.20	0.35	0.12	0.12
LSD	0.39*	0.44*	15.95*	26.87*	0.57*	0.96*	0.34**	0.23**
**B = Zinc rates**								
Zn_1_ = 5 kg ha^–1^	4.95b	5.38b	261.7b	291.9b	9.89c	10.57c	13.52b	13.81c
Zn_2_ = 10 kg ha^–1^	5.21a	5.64a	280.02a	308.18a	11.73c	12.21a	15.36a	16.18a
Zn_3_ = 15 kg ha^–1^	5.00b	5.50b	271.9ab	303.7ab	10.65b	11.17b	15.33a	15.35b
SE	0.09	0.06	4.94	5.97	0.31	0.23	0.09	0.18
LSD	0.20*	0.12**	10.77*	13.02*	0.68*	0.51*	0.19**	0.38**
**C = Nitrogen rates**								
N_1_ = 70 kg ha^–1^	4.85b	5.41b	257.54c	295.13c	10.25b	10.81c	15.58b	14.88b
N_2_ = 140 kg ha^–1^	5.34a	5.57a	285.29a	307.76a	11.13a	11.82a	14.92a	15.30a
N_3_ = 210 kg ha^–1^	4.98b	5.54a	270.8b	300.8b	10.88a	11.32b	14.70ab	15.17a
SE	0.08	0.05	4.08	2.63	0.14	0.15	0.14	0.10
LSD	0.20**	0.12*	8.27**	5.34*	0.29**	0.31**	0.27*	0.21**
**Interactive effect of irrigation (A), zinc (B), and nitrogen (C) rates**								
A × B	NS	**	NS	NS	NS	NS	**	NS
A × C	NS	NS	NS	NS	NS	NS	NS	NS
B × C	*	**	NS	NS	NS	NS	NS	NS
A × B × C	NS	NS	NS	NS	NS	NS	NS	NS

Means sharing the same alphabet within the column are not statistically significant.

SE, standard error; LSD, least significant difference value for comparison; NS, statistically non-significant.

* and ** Significant at P < 0.05 and 0.01, respectively.

Data regarding net assimilation rate (NAR), photosynthetically active radiation (PAR), radiation use efficiency (RUE) with respect to total dry matter (TDM), and kernel yield (KY) are presented in Table 6. Highest increment in all the said parameters was observed in the case of 12-acre inches irrigation level. Maximum values of NAR, PAR, RUE to TDM and RUE to KY were observed in 12-acre inches, while minimum values were attained in 8-acre inches irrigation level at both sowing places (Table 6). Regarding Zn application, 10 kg ha^–1^ was more responsive in aforesaid attributes and has attained maximum values, while lowest values were observed in case of 5 kg ha^–1^ Zn application at both sowing places. In the case of N, 140 kg ha^–1^ was more responsive compared to other N rates at both sowing places. Lowest values of attributes presented in Table 6 were observed where 70 kg ha^–1^ N was supplemented. Interactive effect between irrigation levels and Zn and N application was found non-significant (Table 6).

**TABLE 6:** Impact of zinc and nitrogen rates on net assimilation rate (NAR), photosynthetically active radiation (PAR), and radiation use efficiency (RUE) with respect to total dry matter (TDM) and kernel yield (KY) of rice cultivated under three irrigation levels at Sheikhupura (D1) and Sargodha (D2) districts of Pakistan.

Parameters	NAR		PAR		RUE to TDM		RUE to KY	
				
Treatments	D1	D2	D1	D2	D1	D2	D1	D2
**A = Irrigation levels**								
I_1_ = 8	5.51c	4.97b	826.41b	873.29b	1.73b	1.65b	0.66b	0.57c
I_1_ = 12	5.72a	5.44a	861.72a	910.57a	1.82a	1.81a	0.71a	0.61a
I_1_ = 16	5.59b	5.18ab	846.86a	890.03ab	1.77ab	1.73ab	0.68b	0.58b
SE	0.02	0.13	7.06	9.05	0.02	0.04	0.01	0.01
LSD	0.06*	0.35*	19.61*	25.13*	0.06*	0.10*	0.03*	0.02*
**B = Zinc rates**								
Zn_1_ = 5 kg ha^–1^	5.38b	5.05b	829.95b	880.62b	1.68b	1.65b	0.65b	0.54c
Zn_2_ = 10 kg ha^–1^	5.85a	5.42a	857.79a	897.36a	1.88a	1.83a	0.70a	0.65a
Zn_3_ = 15 kg ha^–1^	5.59ab	5.11ab	847.25a	895.91a	1.76b	1.71b	0.69a	0.58b
SE	0.22	0.16	7.02	5.60	0.04	0.04	0.03	0.02
LSD	0.47*	0.34*	15.29*	12.21*	0.09**	0.80**	0.03*	0.04*
**C = Nitrogen rates**								
N_1_ = 70 kg ha^–1^	5.41b	5.06b	826.66c	884.67b	1.70c	1.67c	0.65b	0.57b
N_2_ = 140 kg ha^–1^	5.75a	5.36a	863.33a	901.72a	1.83a	1.81a	0.71a	0.62a
N_3_ = 210 kg ha^–1^	5.66a	5.16b	845.00b	887.50b	1.79b	1.71b	0.68a	0.57b
SE	0.09	0.08	6.31	3.02	0.02	0.02	0.02	0.01
LSD	0.18**	0.16**	12.79**	6.12**	0.03**	0.04**	0.03*	0.02*
**Interactive effect of irrigation (A), zinc (B), and nitrogen (C) rates**								
A × B	NS	NS	NS	NS	NS	NS	NS	NS
A × C	NS	NS	NS	NS	NS	NS	NS	NS
B × C	NS	NS	NS	NS	NS	NS	NS	NS
A × B × C	NS	NS	NS	NS	NS	NS	NS	NS

Means sharing the same alphabet within the column are not statistically significant.

SE, standard error; LSD, least significant difference value for comparison; NS, statistically non-significant.

* and ** Significant at P < 0.05 and 0.01, respectively.

## Discussion

Water consumption in agriculture sector is increasing day by day while its resources are declining, so using irrigation water for rice production in areas of high-water stress can be a challenging issue. Water requirement for rice irrigation needs continuous flooding to maximize kernel yields (Du et al., 2022). In fact, without full immersion, weeds are difficult to control, and yield is adversely affected. Water quality has a significant impact on rice growth, yield characteristics, and yield (Prasad et al., 2008; Rezaei et al., 2013). The results of present experimentation showed that increasing the irrigation level significantly increased the TDM compared to the lower irrigation level. However, the results also showed that the difference between 12-acre inches and 16-acre inches was not significant. These findings are supported by Mukherjee and Mandal (1995), who conducted a field trial to study the effect of full immersion and nitrogen fertilization on rice yield. They observed that flooding conditions were positively correlated with grain yield and nitrogen yield. Similarly, Thanunathan and Sivasubramanian (2002) showed that the highest number of high-density grains was recorded in continuous submergence. Sharma et al. (2008) concluded that pre-sowing irrigation and gradual fertilization have a positive effect on straw yield and nutrient content of straw crops. Similarly, increasing Zn application levels also increased TDM, a response commonly seen in Punjab rice (Ahmad et al., 2009). Therefore, irrigation and Zn fertilization possibly increase TDM yield due to the increase in LAI, LAD, and, hence, CGR (Ahmad et al., 2009). These results also confirmed that under the current environmental and soil conditions, 12-acre inches and 10 kg Zn ha^–1^ were sufficient to achieve higher TDM. The amount of water needed to water plants depends on climate, plant, and soil conditions. However, water requirements can be modified by changing irrigation schedules and cultivation methods (Kim et al., 1992; Sharma and Sarkar, 1994). A study in Punjab (Pakistan) (Ahmad et al., 2009) compared five irrigation patterns (62.5, 77.5, 92.5, 107.5, and 1,122.5 cm) with Basmati-385 cultivar of basmati rice.

Nitrogen (N) plays a key role in plant growth in intensive agriculture as an essential component of cellular components. Therefore, insufficient nitrogen intake has profound effects on food production. Some early researchers reported that the rate and timing of nitrogen application played an important role in improving the yield of rice and other field crops (Habtergebrial et al., 2013; Khan et al., 2021). These authors found the highest yields (4–5-ton ha^–1^) of basmati rice and its components at 130–140 kg N ha^–1^. The outcomes of current experimentations are in line with the results of Rajarathinam and Balasubramaniyan (1999) who found that the components of rice yield (such as number of ears m^–2^, number of ears^–1^, and thousand kernel weight), rice yield, and index yield were highest and amounted to 200 kg N ha^–1^. Nitrogen is an integral part of protein, an important component of protoplasm and enzymes, and a biological catalyst that accelerates the life process. It is also an important component of organic compounds, such as core proteins, amino acids, amines, amino sugars, and plant polypeptides. Iqbal et al. (2020) reported that application of farmyard manure was responsible to improve the growth of maize crop. Nitrogen fertilization significantly affects the yield of super hybrid rice (Zheng et al., 2008; Yoseftabar et al., 2012). Kavitha et al. (2008) concluded that using 200 kg N ha^–1^ improves nutrient uptake, yield, and economics of rice. Grain yield and shoot dry matter increased significantly with increasing nitrogen fertilization in the range of 0–200 kg ha^–1^ (Fageria et al., 2008). Application of plant growth enhancers, including mineral elements, promotes the stand establishment, and crop yield is ultimately improved under normal and stress full conditions (Rehman et al., 2021; Koleva et al., 2022).

The emergence of high-yielding varieties, characterized by a better response to nitrogen, requires appropriate nitrogen fertilizers since it becomes necessary to increase their resistance to flooding in flood conditions. The main obstacle to the use of nitrogen under such uncontrolled hydrological conditions is the low yield, which rarely exceeds 25–30% (Mohanty et al., 1999). Higher N doses (210 kg ha^–1^) did not lead to higher TDM yields compared to lower N doses (140 kg ha^–1^). This may be due to the large losses of N and NH_3_ volatilization under submerged conditions treated with higher doses of N. Nutrient losses from paddy fields can be high, and during periods of heavy rain, the flow of water from field to field not only reduces nutrient use efficiency but can also lead to environmental degradation (Kapoor et al., 2008). Mineral fertilization significantly enhanced the yield of field crops, including rice (Khasanah and Rachmawati, 2020; Tanga et al., 2020). Ahmad et al. (2009) also reported higher TDM yields in basmati rice with increasing irrigation levels and nitrogen application rates. The increase in rice yields due to the use of Zn was consistent with the previous report. The higher yield has been attributed to factors, such as the number of shoots on the mound and the weight of a thousand stones. Seasonal plant growth was determined by DM accumulation and leaf area development at both sites. The effect of N and Zn treatment on TDM was significant, but there were differences between different harvest dates. Irrigation levels showed no significant response at all harvest times. Ahmad et al. (2009) reported a similar growth curve for TDM accumulation in basmati rice, working under similar agroclimatic conditions. Monteith and Elston (1983) argued that the relationship between yield and LAD is not causal because LAD does not account for differences in light trapping, leaf shading, tree canopy structure, and rates of photosynthesis. Therefore, if the LAI exceeds, no additional radiation disruption occurs, and this additional LAI provides no benefit to the crop. Nitrogen fertilization plays critical roles in physiomorphological traits of field crops, which are directly and/or indirectly linked with economical yield (Kumar et al., 2022).

The mean crop growth rate (CGR) was affected by increased doses of zinc and nitrogen at both sites. However, the difference in mean CGR between irrigation levels was not significant. The average CGR values for Basmati rice reported here compare favorably to those from other regions. Ahmad et al. (2009) reported mean CDRs ranging from 12 to 14.50 g/m^2^ day^–1^ at the Faisalabad, Kala Shah Kaku, and Gujranwala sites. The results showed that increasing zinc and nitrogen doses significantly improved the efficacy of CT irradiation (RUE). Averaging these two sites, Sheikhupura and Sargodha had ERU averages of 1.77 and 1.73 g MJ^–1^, respectively. Similar dependencies have been reported by others (Ahmad et al., 2009) who also reported ERU values ranging from 1.41 to 1.44 g MJ^–1^. Data on quality parameters of transplanted fine rice affected by increased rehydration, zinc, and nitrogen levels showed significant but quadratic responses for all treatments. The results presented in this experiment generally show that higher doses of irrigation, zinc, or nitrogen application decrease the appearance of most grades. Similar results have been reported by others (Ahmad et al., 2009). Treatments, such as slower watering rates, zinc, and nitrogen consumption, may be too low to cause nutritional stress, which can increase grain size reduction. Shivay et al. (2008) and Khan et al. (2009) reported that using zinc in an amount of 10–15 kg ha^–1^ can improve the yield and quality of milled rice.

## Conclusion

In an intensive rice production system, rice productivity is significantly influenced by several factors, including low plant population, irrigation water shortage, zinc deficiency, imbalanced use of fertilizers, and inadequate plant protection measures. Experiments were conducted to examine the impact of three levels of zinc and nitrogen under three irrigation regimes on the growth and quality of rice crop. The outcomes of current experimentations showed that application of irrigational water, zinc, and nitrogen at 12-acre inches, 10, and 140 kg ha^–1^, respectively, are responsible to achieve maximum kernel yield and quality of produce. Further studies regarding integrated application of macro and micronutrients are required to enhance the resources use efficiency with improved crop productivity.

## Data availability statement

The original contributions presented in the study are included in the article/supplementary material, further inquiries can be directed to the corresponding authors.

## Author contributions

All authors actively participated in designing and conducting the experiment, data collection, statistically analysis, and manuscript preparation.
